# Cleaning Efficiency of Root Canal after Irrigation with New Irrigation Technique: A Scanning Electron Microscopic Study 

**DOI:** 10.22037/iej.v13i1.17285

**Published:** 2018

**Authors:** Linz A. Shalan, Hussain F. Al-huwaizi

**Affiliations:** a *Department of Conservative and Esthetic Dentistry, College of Dentistry, University of Baghdad, Baghdad, Iraq*

**Keywords:** AquaPick Device, Endoactivator, Smear Layer Removal, Pressurized Water

## Abstract

**Introduction:**

The aim of this study was to evaluate the ability of pressurized water irrigation technique (AquaPick Device) as an intra-canal irrigation technique and compare it with sonic irrigation device (Endoactivator) for their ability to remove smear layer from canals.

**Methods and Materials:**

Total number of 80 single rooted teeth (premolars) were prepared, divided into eight main groups, Group 1: Aquapick with apically vented needle/18 mm depth, Group 2: Aquapick with apically vented needle/15 mm depth, Group 3: Endoactivator device/18 mm depth, Group 4: Endoactivator device/15 mm depth, Group5: Aquapick with 2 side vented needle/18 mm depth, Group 6: Aquapick with 2 side vented needle/15 mm depth and two control groups. Then all samples were tested by SEM in 3, 6 and 9-mm distances from the apical foramen. The data were statistically analyzed using Kruskal Wallis and Mann-Whitney U tests.

**Results:**

There was a high significant difference among the tested groups with the best removal of smear layer by the use of pressurized water irrigation device with apical vented needle especially at the 3 mm area**. **

**Conclusion:**

Pressurized water irrigation technique could be used as intra-canal irrigation technique with good results.

## Introduction

The goals of root canal therapy are to remove infected and necrotic pulpal tissues, shape the root canal system and provide adequate sealing using obturation materials [1]. Pulpal tissue remnants will stay attached to dentine walls even with current cleaning and shaping techniques. The inner configuration of the root canal system and the pulpal space are highly complex [2]. There are lateral and accessory canals that make root canal treatment even more difficult. Therefore, there is a need for appropriate instruments and irrigants for chemo mechanical instrumentation of the root canal system.

For successful root canal treatment, a system that delivers the irrigant effectively to the working length is required. Conventional irrigation with needles is the standard procedure but is not effective in apical third of the root canal and difficult anatomy of the apical zone [3, 4]. These irrigants must be brought into direct contact with the entire canal wall for effective action. During conventional needle irrigation, replenishment and fluid exchange do not extend much beyond the tip of the irrigating needle [5]. That is why different techniques and irrigant delivery devices have been proposed to increase the flow and distribution of irrigating solutions within the root canal system [6].

The smear layer consists of dentin, remnants of odontoblastic processes, pulp tissue and bacteria [7]. The smear material is divided into two parts: First, superficial smear layer and second, the material packed into the dentinal tubules. Packing of smear debris may be present in the tubules to a depth of 40 μm.

The Endoactivator system (Dentsply Sirona, GmbH, Bensheim, Germany) can be used to improve the efficiency of irrigation. Mechanical oscillations are produced mainly at the tip of the plastic activator with a frequency ranging from 2 to 10 kHz [8].

**Figure 1:**
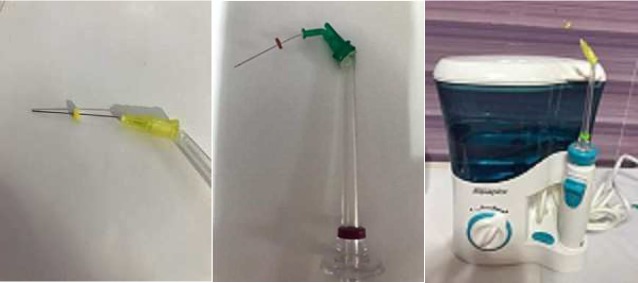
Irrigation devices

Aquapick AQ-300 device (Aquapick Co, Ltd, Korea) is present in markets as an advanced oral irrigation device with 1800 pulsations per minimum and maximum water pressure is 7kgf/cm.

The aim of this study was to evaluate the ability of pressurized water irrigation device (Aquapick) to be used as intra-canal irrigation device after some modifications and compare it with sonic irrigation device (Endoactivator) for their ability to remove smear layer from apical, middle and cervical thirds of root canal.

## Materials and Methods

***Preparation of the samples***

Eighty permanent human single rooted teeth were selected according to the following criteria: single canal with mature apices of the roots, no root caries or resorption, patent apical foramen in which size 10 file should pass through the apex without any resistance and size 15 file cannot pass easily. The exclusion criteria used in this study were the following: No cracks in the roots of the teeth. Then teeth were cleaned with cumin scaler to remove calculus and soft tissue debris then washed under tap water and kept in distilled water solution [9]. 

The teeth were divided into eight experimental groups (*n*=10); each group containing similar numbers of the same tooth types with similar canal length, To ensure that the cleaning efficiency was due to the irrigation technique and not to tooth morphology or irrigants type [10]. The teeth were forced through a precut hole in a rubber stopper, then placed on the glass shell vials. A 27-gauge needle (KDL-China) was placed through the stopper into the flask to equalize the air pressure inside and outside the vial [10].

Access opening was made to the teeth then working length was determined by placing #10 K-file (Dentsply, Maillefer, Ballaigues, Switzerland) with a rubber stop carefully inserted into each canal until it was just visible in the apical foramen. This length was noted and 1 mm was subtracted to give the working length of the canal and all the selected teeth had a 19-mm working length. The teeth were prepared with ProTaper hand system (Dentsply, Maillefer, Ballaigues, Switzerland) in crown-down approach and the instruments were used in sequence recommended by manufacturer’s instructions and used to enlarge five canals only. The apical enlargement was prepared to size F2 (D0=25) [11]. Each time 4 mL of distilled water was used as irrigant with a duration of 30 sec [12] after each file with total irrigation time of 120 sec. Five samples were instrumented at a time to minimize operator fatigue.

**Figure 2:**
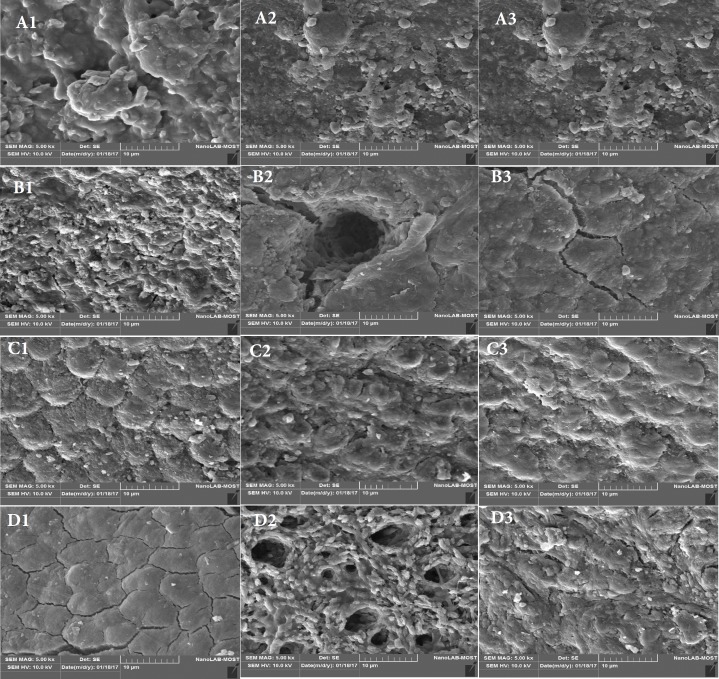
*A)* SEM of group 1 at 5000× magnification: *a.* 3mm, *b.* 6mm, *c.* 9mm; *B)* SEM of group 2 at 5000× magnification: *a.* 3mm, *b.* 6mm, *c.* 9mm; *C) *SEM of group 3 at 5000× magnification: a. 3mm. b. 6mm, *c.* 9mm; *D*) SEM of group 4 at 5000× magnification: *a*. 3mm. *b*. 6mm. *c*. 9mm

For pressurized water, the Aquapick device was modified by the addition of dental needle gauge 23 (apically vented) to its tip (Figure 1A) and 2 side vented syringe gauge 23 (Figure 1B) added to another tip as shown in Figure 1C.

***Grouping***

A total of 80 freshly extracted single rooted teeth (permanent premolars) were used in this study which was divided into 8 groups (n=10) as follows: Group 1: Aquapick + apically vented needle inserted 18 mm inside canal, Group 2: Aquapick + apically vented needle inserted 15 mm inside canal, Group 3: Irrigation by Endoactivator device inserted 18 mm inside canal, Group 4: Irrigation by Endoactivator device inserted 15 mm inside canal, Group 5: Aquapick + 2 side vented needle inserted 18 mm inside canal, Group 6: Aquapick + 2 side vented needle inserted 15 mm inside canal, Group 7: Hypodermic syringe and 23 gauge needle inserted 18 mm inside canal (control group) and Group 8: Hypodermic syringe and 23 gauge needle inserted 15 mm inside canal (control group). 

**Table 1:** Scores for groups & subgroups, Kruskal Wallis test and Mann-Whitney U test

	**3 mm**	**6 mm**	**9 mm**	**Kruskal Wallis Test**	**Significance **	**Mann-Whitney U**	**Significance **
**Aquapick 18mm**	3	3	3	0.513	NS	-------	-------
**Aquapick 15mm**	4	3	2				
**Endoactivator 18mm**	5	3	3	0.007	HS	0	HS
**Endoactivator 15mm**	4	1	3				
**Aquapick 18mm**	5	2	2	0.006	HS	0	HS
**Aquapick 15mm**	5	3	3				
**hypodermic syringe 18mm**	5	5	5	0.311	NS		
**hypodermic syringe 15mm**	5	4	4				

***SEM evaluation***

Root canals of each tested group were dried with paper points [13] and the casting wax sealing the apical foramen of each root was removed. Roots were split longitudinally in a bucco-lingual direction to expose root interior by making two grooves on the buccal and lingual aspects of each root with a low speed diamond disk. The grooves were not deep enough to enter the canals and a plastic instrument was then used to section the root into two halves [12]. For each root, the half containing the most visible part of apex was conserved and coded. Roots showing evidence that the grooves had penetrated into the root canal or exhibiting an irregular cleavage were discarded and replaced by new specimens.

Coded samples were mounted on metallic stubs, sputter gold-coated to render the surface electrically conductive, and then examined under SEM under ×5000 magnification. Three pictures were obtained from each tooth, one for each third, to give a total of 240 pictures at (3 mm, 6 mm and 9 mm from the apical foramen, respectively) [14]. The images were analyzed for the amount of smear layer. Mayer *et al*. [15] scored as: 1, no smear layer; 2, few areas covered by smear layer with many dentin tubule orifices visible; 3, most areas covered by smear layer, with few dentin tubule orifices visible; 4, all areas covered by smear layer, no dentin tubule orifices visible; 5, Heavy, non-homogeneous smear layer covering the complete root canal wall.

**Figure 3:**
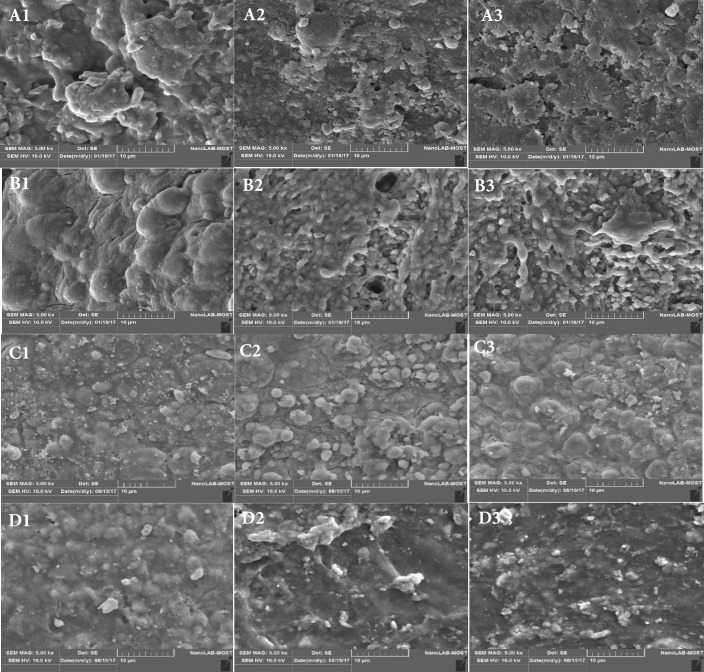
*A)* SEM of group 5 at 5000× magnification: A. 3mm. B. 6mm. C. 9mm; *B)* SEM of group 6 at 5000× magnification: *a.* 3mm, *b.* 6mm, *c.* 9mm; *C) *SEM of group 7 at 5000× magnification: a. 3mm. b. 6mm, *c.* 9mm; *D*) SEM of group 8 at 5000× magnification: *a*. 3mm. *b*. 6mm. *c*. 9mm

The data were statistically analyzed, by using Kruskal Wallis and Mann-Whitney U test. The level of significance was set at 0.05.

## Results

On analysis of the cleanliness using scanning electron microscopy, for the various groups are reported in Table 1 as scores.

At 3 mm from the apex, the dentin surface was covered by heavy coherent deposits of smear layer and debris with irregular shapes and sizes, and the dentinal tubules were not visible in all groups, with the exception of tooth irrigated with Aquapick with apical vented needle to 18 mm which scored 3 while Aquapick with apical vented needle to 15 mm, and Endoactivator to 15 mm both had score 4 when compared with control group with high significant differences.

At 6 mm from the apex, groups showed statistically high significant differences (*P*>0.5) when compared with the control group with score 1 for Endoactivator to 15 mm, Aquapick with 2 side vented needle to 18 mm score 2, the rest of groups had score 3.

At 9 mm from the apex, groups showed statistically high significant differences (*P*>0.5) when compared with the control groups with score 2 for Aquapick with apical vented needle to 15mm, Aquapick with 2 side vented needle to 18 mm score 3 for the rest of the groups (Figures 2 and 3).

## Discussion

The present study focused on the ability of pressurized water on removal of smear layer during root canal preparation with 2 types of dental needles.

Penetration depth of the irrigation needle affects irrigant extrusion and apical needle placement improves cleaning and disinfection [16] . Aqua-pick device with apically vented needle and insertion of the tip of irrigation device to (15 mm inside the canal) showed high significant (*P*< 0.5) differences between groups with less efficient removal of smear layer at 3 mm-area while in the 6 mm-area is equal and in 9 mm-area was better. This is may be related to the fact that the irrigation solution was delivered in the middle and apical region did not have sufficient irrigant movement to aid in cleaning this area. These results are in agreement with the results reported by Cheung and Stock in 1993 [17].

Endoactivator sonic device had high significant difference between the groups which may be because the tip of the Endoactivator is far from apical region which already has limited space to agitate so there was no effect on smear layer. Maximum tip agitation was in middle area so cleaning was best there. The 9 mm-area had moderate cleaning therefore insertion of 15 mm inside canal can produce better smear layer removal in that area and this findings are in agreement with other study[18]. 

Aquapick with 2 side vented needle had high significant differences at depth of 18 mm. The result was related to the fact that at the apical region, pressure is distributed through 2 openings so cleaning is less but the 2 openings make irrigation acoustic streaming of the irrigant better so the 6 mm-area and 9 mm-area showed better removal of the smear layer. At a depth of 15 mm, the result related to pressure of double vented needle distributed to two openings and as the tip is 4 mm from apex there was no effect on smear layer. The 6and 9 mm-areas had more copious irrigant so moderate removal of smear layer.

The double side-vented group produced cleaner canals at the 9 mm-area and 6 mm-area compared to the apically vented group, which is in agreement with many studies [19, 20] that showed the perforated endodontic irrigation needles had a greater distribution of irrigating solution and cleaner canals than a conventional irrigation needle. 

Aquapick device had water pressure which is 7 kgf/cm and 1800 pulsation per min which produces vacuum inside the canal which lead to better cleaning efficiency. For Endoactivator, the oscillating patterns of the sonic instruments are different. They have one node near the attachment of the file and one antinode at the tip of the file [3]. It generates acoustic streaming of the irrigant that removes the smear layer also the cleaning efficiency results for Endoactivator in apical area in this study could be explained as the tip reaches the apical region the space needed for the tip agitation is decreased therefore less cleaning of the root canal wall is expected [18]. There is no literature about using pressurized water in root canal irrigation so further researches are required.

## Conclusion

Within the limitation of this study the pressurized water could be used as intra-canal irrigation technique with good cleaning efficiency especially at the apical third with low coast when compared with Endoactivator system.
